# Effects of a 7-day military training exercise on inflammatory biomarkers, serum hepcidin, and iron status

**DOI:** 10.1186/1475-2891-12-141

**Published:** 2013-11-04

**Authors:** James P McClung, Svein Martini, Nancy E Murphy, Scott J Montain, Lee M Margolis, Ingjerd Thrane, Marissa G Spitz, Janet-Martha Blatny, Andrew J Young, Yngvar Gundersen, Stefan M Pasiakos

**Affiliations:** 1 Military Nutrition Division, US Army Research Institute of Environmental Medicine, Natick, MA, USA; 2 Norwegian Defence Research Establishment, Kjeller, Norway; 3 Thermal Mountain and Medicine Division, US Army Research Institute of Environmental Medicine, Natick, MA, USA

**Keywords:** Physical activity, Operational stress, Military, Ferritin, Inflammation, Iron absorption, Soluble transferrin receptor

## Abstract

**Background:**

Hepcidin, a peptide that is released into the blood in response to inflammation, prevents cellular iron export and results in declines in iron status. Elevated serum and urinary levels of hepcidin have been observed in athletes following exercise, and declines in iron status have been reported following prolonged periods of training. The objective of this observational study was to characterize the effects of an occupational task, military training, on iron status, inflammation, and serum hepcidin.

**Findings:**

Volunteers (n = 21 males) included Norwegian Soldiers participating in a 7-day winter training exercise that culminated in a 3-day, 54 km ski march. Fasted blood samples were collected at baseline, on day 4 (PRE, prior to the ski march), and again on day 7 (POST, following the ski march). Samples were analyzed for hemoglobin, serum ferritin, soluble transferrin receptor (sTfR), interleukin-6 (IL-6), and serum hepcidin. Military training affected inflammation and serum hepcidin levels, as IL-6 and hepcidin concentrations increased (*P* < 0.05) from the baseline to POST (mean ± SD, 9.1 ± 4.9 vs. 14.5 ± 8.4 pg/mL and 6.5 ± 3.5 vs. 10.2 ± 6.9 ng/mL, respectively). Iron status was not affected by the training exercise, as sTfR levels did not change over the course of the 7-day study.

**Conclusions:**

Military training resulted in significant elevations in IL-6 and serum hepcidin. Future studies should strive to identify the role of hepcidin in the adaptive response to exercise, as well as countermeasures for the prevention of chronic or repeated elevations in serum hepcidin due to exercise or sustained occupational tasks which may result in longer term decrements in iron status.

## Findings

### Introduction

The relationships between iron status and physical and neuropsychological performance in humans are well established and relate to the biological action of iron dependent proteins and enzymes [1,2]. Examples include hemoglobin and myoglobin, which support oxygen storage and transport, and aconitase and cytochrome c, which function in energy metabolic pathways. Maintaining optimal iron status is paramount for athletes and individuals with physically demanding occupations, such as military service, as poor iron status has been linked to diminished performance in such individuals [3,4], and iron status may decline in response to sustained physical activity [4-6].

Several mechanisms have been proposed to explain declines in iron status associated with physical activity, to include gastrointestinal bleeding [7] and iron losses in sweat [8] and urine [9]. The biological activity of hepcidin, a 25-amino acid protein that arises in response to proinflammatory cytokines, including interleukin-6 (IL-6), may represent another mechanism by which iron status declines in response to physical activity [10]. Hepcidin affects iron export from the enterocyte, macrophage, and hepatocyte through binding of ferroportin 1 (FPN1), the primary cellular iron export protein [11]. The binding of hepcidin to FPN1 results in the degradation of the hepcidin-FPN1 complex, effectively sequestering iron, thereby limiting availability for incorporation into iron-dependent proteins and enzymes.

Recent studies have investigated the effects of endurance-type exercises, such as running and cycling, on serum hepcidin and inflammatory biomarkers in athletes. For example, Newlin et al. [12] observed significant elevations in serum hepcidin following treadmill running in female athletes, and Sim et al. [13] observed elevated IL-6 and serum hepcidin in male triathletes following both running and cycling activities. However, the effects of more complex occupational tasks, such as short-term military training, on hepcidin levels and iron status have not been investigated. The objective of this observational study was to characterize the effects of a 7-day winter military training exercise on iron status, biomarkers of inflammation, and serum hepcidin.

## Methods

### Participants

Norwegian Soldiers (n = 21 males) from the Garrison in Sør-Varanger (Kirkenes, NO) volunteered for this study. The Soldiers were participating in a 7-day winter training exercise that culminated in a 3-day, 54 km ski march along the Norwegian-Russian border (Table 1). Indicators of iron status, serum hepcidin, and IL-6 concentrations were assessed at baseline, following a 4-day pre-march training period (PRE), and immediately after completing a 3-day ski march (POST). The Human Use Review Committee at the U.S. Army Research Institute of Environmental Medicine (Natick, MA, USA) and the Regional Committees for Medical and Health Research Ethics (REK sør-øst, Oslo, NO) approved this study.

**Table 1:** Volunteer characteristics

**Age (y)**	**20 ± 1**
Height (cm)	182 ± 7
Weight (kg)	82 ± 9
BMI (kg/m^2^)	25 ± 2

Data are means ± SD (n = 21). BMI, body mass index.

### 7-Day winter training exercise

The training exercise was conducted in March 2013 when mean outdoor temperature was -15 ± 4°C (low -26°C and high -6.2°C). After baseline data collection (morning day 0), Soldiers conducted 4 days (days 0–3) of military-specific training exercises, including weapons training, mountainous terrain navigation, and general winter survival training. After completing PRE data collection (morning day 4), Soldiers began the 3-day (days 4–6) cross-country ski march, skiing in 50:10 min work to rest ratios for 6–10 hours per day, travelling nearly 20 km per day, while carrying ~45 kg of additional gear. Soldiers subsisted solely on Norwegian arctic combat rations during the 7-day exercise. Soldiers were provided three rations during the 4-day pre march, and four rations per day during the march (Table 2), although only 66% of the rations were consumed during the march, which may have resulted in a caloric deficit, contributing to the physiological strain of the training exercise.

**Table 2:** Dietary intake

	**Energy (kcal)**	**Protein (g)**	**Carbohydrate (g)**	**Fat (g)**
**Provided**				
PRE	3842 ± 153	161 ± 13	425 ± 20	160 ± 10
Ski March	5177 ± 325	213 ± 13	572 ± 19	218 ± 41
**Consumed**				
PRE	3115 ± 437	128 ± 22	360 ± 57	122 ± 19
Ski March	3415 ± 977	139 ± 42	384 ± 125	138 ± 38

Data are means ± SD (n = 21). Three Norwegian arctic combat rations were provided during the pre-march training (PRE) and four were provided during the ski-march.

### Biological analyses

Blood samples were collected after an overnight fast (Vacutainer, Becton Dickson, Franklin Lakes, NJ). Serum was frozen and shipped to the Pennington Biomedical Research Center (Baton Rouge, LA) for analysis of ferritin (Siemens Medical Solutions USA Inc, Malbern, PA), soluble transferrin receptor (sTfR, Quantikine IVD; R&D Systems Inc, Minneapolis, MN), hepcidin (DRG International, Inc, Springfield, NJ) and IL-6 (Milliplex MAP; Millipore, Billerica, MA). Hemoglobin was assayed in whole blood at the Kirkenes Hospital (NO) using an automated hematology analyzer (Sysmex® XN-Series, Sysmex Norge, Oslo, NO). Multiple indicators of iron status and inflammation were incorporated into the biochemical panel utilized in this study, as alterations in hydrations status are known to affect the interpretation of hematology data.

### Statistical analysis

Baseline volunteer characteristics were described using common descriptive statistics. Shapiro-Wilk tests confirmed data were normally distributed. Repeated measures ANOVAs were used to evaluate changes over time for circulating indices of iron status, hepcidin, and IL-6. Post hoc analyses were completed using Bonferroni corrections. Correlations were assessed using Pearson’s product–moment correlations. Significance was set at *P* < 0.05. Data were analyzed using SPSS (version 19.0; SPSS Inc., Chicago, IL) and expressed as means ± SD.

## Results

Ferritin increased in response to training, as concentrations were approximately 25% higher (*P* < 0.05) PRE and POST as compared to baseline (Table 3). Ferritin levels were similar (*P* > 0.05) at the PRE and POST timepoints. Hemoglobin remained constant (*P* > 0.05) from baseline to PRE but decreased (*P* < 0.05) approximately 4% in response to the 3-day ski-march (POST). Serum sTfR levels did not change over time.

**Table 3:** Changes in iron status during a 7-day winter military training exercise

	**Baseline**	**PRE**	**POST**	**Effect**
Hemoglobin (g/dL)	14.7 ± 0.8^a^	14.5 ± 0.9^a^	14.1 ± 0.9^b^	T
Ferritin (ng/mL)	109.2 ± 44.1^a^	137.0 ± 54.5^b^	133.0 ± 55.2^b^	T
sTfR (nmol/L)	18.9 ± 4.2^a^	18.7 ± 3.8^a^	18.7 ± 3.2^a^	
Hepcidin (ng/mL)	6.5 ± 3.5^a^	6.8 ± 4.1^a^	10.2 ± 6.9^b^	T
IL-6 (pg/mL)	9.1 ± 4.9^a^	7.9 ± 3.9^a^	14.5 ± 8.4^b^	T

Data are means ± SD (n = 21). sTfR, soluble transferring receptor; IL-6, interleukin 6. T, main effect of time determined using repeated-measures ANOVA with post-hoc Bonferroni corrections for multiple comparisons. Means not sharing the same superscript are different, *P* < 0.05.

Serum hepcidin remained constant (*P* > 0.05) from baseline to PRE, yet concentrations increased (*P* < 0.05) nearly 57% in response to the 3-day ski march (POST, Table 3). Similarly, IL-6 concentrations were steady (P > 0.05) from baseline to PRE but were approximately 60% higher (*P* < 0.05) POST (Table 3). Significant (P < 0.05) associations were identified between POST serum hepcidin, IL-6 (*r* = 0.6) and ferritin (*r* = 0.5).

## Discussion

The major finding of this longitudinal, repeated measures study was that a 7-day winter military training activity resulted in significant elevations in IL-6 and serum hepcidin. This is the first report of elevated IL-6 and serum hepcidin in response to an applied occupational task, and may raise the possibility that repeated exposures to strenuous occupational tasks could degrade iron status, which could eventually affect physical and neuropsychological performance over time.

Findings from the present study are consistent with previous studies in athletes. For example, Roecker et al. [14] reported elevated urinary hepcidin in female athletes following a marathon. Subsequently, Newlin et al. [12] reported elevated IL-6, serum ferritin, and hepcidin in response to treadmill running (60 and 120 mins at 65% of maximal oxygen consumption) in female volunteers. Similarly, Sim et al. [13] reported elevated levels of IL-6 and serum hepcidin following low intensity steady-state running (40 mins at 65% of peak running velocity), low intensity steady-state cycling (40 mins at 65% of peak cycling power output), high-intensity interval running (8 × 3 min intervals at 85% of peak running velocity) and high-intensity interval cycling (8 × 3 min intervals at 85% of peak cycling power output) in male triathletes. Similar to Newlin et al. [12] and Sim et al. [13], both serum ferritin and hepcidin were elevated in response to physical activity. However, in the present study, the increase in serum ferritin (PRE) preceded the increase in hepcidin (POST), suggesting that ferritin could be more sensitive to immediate changes in inflammation (or occur more rapidly) than hepcidin, although further study would be required to directly address this hypothesis.

In the present study, hepcidin was detected using a commercially available kit (DRG International). Others have used different techniques for hepcidin analysis, including a combination of weak cation exchange chromatography and time-of-flight mass spectrometry [15]. The use of multiple techniques across laboratories makes comparison of absolute levels of hepcidin difficult, although the repeated measures design of the current study provides confidence in the relative change in hepcidin concentrations over time.

Although the military training exercise had a significant effect on inflammation and serum hepcidin, iron status was unchanged. sTfR, the gold-standard iron status indicator, was unaffected by the exercise, and hemoglobin declined minimally (4%). Serum ferritin was elevated in response to the training exercise, probably reflecting the effects of acute inflammation, and not improved iron status [16]. The lack of change in iron status may reflect the duration of data collection, as decrements may have become apparent if subsequent samples were collected. Since the male Soldiers that participated in this training exercise had robust iron stores at baseline, it is unlikely that decrements in iron status would have an immediate effect on performance. However, individuals that begin similar training exercises with moderate or poor iron status, such as female Soldiers (4, 6, 17), may experience declines in iron status that affect performance, especially if training exercises were extended over longer periods, or followed by other physically demanding, occupational tasks (e.g., operational deployment).

Future studies should focus on identifying the role of hepcidin in the adaptive response to exercise, as well as the efficacy of hepcidin antagonism for the preservation of iron status in individuals that experience repeated bouts of physical activity that may result in an inflammatory response. Work from our laboratory indicates that providing supplemental iron may prevent declines in iron status in women that begin training with poor iron status [4,6,17], although the effects of supplemental iron on serum hepcidin remain understudied. Two studies have assessed the effects of carbohydrate ingestion on IL-6 and serum hepcidin during endurance running, although the inflammatory response to exercise was not affected by carbohydrate ingestion [18,19]. To the best of our knowledge, the effects of anti-inflammatory nutrients or pharmacologic agents on serum hepcidin in response to physical activity have not been determined.

## Conclusions

Occupational tasks, such as sustained, physically demanding military training, may result in the release of IL-6, and subsequent elevations in serum hepcidin. Chronic or repeated elevations in serum hepcidin may result in diminished iron status, which could affect human performance. Future studies should strive to identify the role of hepcidin in the adaptive response to exercise, as well as appropriate countermeasures for the prevention of decrements in iron status that may occur in response to physical activity-induced elevations in serum hepcidin.

## Abbreviations

FPN1: Ferroportin 1; IL-6: Interleukin-6; sTfR: Soluble transferrin receptor

## Competing interests

The authors declare that they have no competing interest.

## Authors’ contributions

The authors’ responsibilities were as follows – SMP, SM, YG, SJM, and AJY designed the research; SMP, SM, NEM, LMM, SJM, IT, MGS, and YG conducted the research; JPM, SMP, and LMM analyzed the data; JPM and SMP wrote the manuscript; all authors had responsibility for final content. All authors read and approved the final manuscript.
